# (*E*)-2-Chloro-*N*′-(4-hy­droxy­benzyl­idene)­benzohydrazide

**DOI:** 10.1107/S1600536812005661

**Published:** 2012-02-17

**Authors:** Xiao-Yan Li

**Affiliations:** a Zibo Vocational Institute, Zibo 255314, People’s Republic of China

## Abstract

The title hydrazone mol­ecule, C_14_H_11_ClN_2_O_2_, has a *trans* conformation with respect to the methyl­idene unit. The dihedral angle between the two benzene rings is 37.6 (3)°. In the crystal, the presence of O—H⋯O, O—H⋯N and N—H⋯O hydrogen bonds leads to the formation of a three-dimensional network. The title compound crystallized in the chiral ortho­rhom­bic space group *P*2_1_2_1_2_1_ and was refined as an inversion twin [Flack parameter = −0.20 (18)].

## Related literature

For the syntheses and crystal structures of hydrazone compounds, see: Hashemian *et al.* (2011 ▶); Lei (2011 ▶); Shalash *et al.* (2010 ▶). For the crystal structures of similar compounds, reported recently by the author, see: Li (2011*a* ▶,*b* ▶).
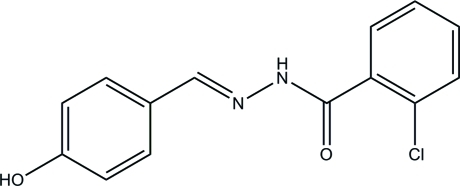


## Experimental

### 

#### Crystal data

C_14_H_11_ClN_2_O_2_
*M* *_r_* = 274.70
Orthorhombic, 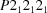

*a* = 7.627 (3) Å
*b* = 11.859 (2) Å
*c* = 14.297 (2) Å
*V* = 1293.2 (5) Å^3^
*Z* = 4
Mo *K*α radiation
μ = 0.29 mm^−1^
*T* = 298 K
0.18 × 0.17 × 0.17 mm

#### Data collection

Bruker SMART CCD area-detector diffractometer
Absorption correction: multi-scan (*SADABS*; Sheldrick, 1996 ▶) *T*_min_ = 0.949, *T*_max_ = 0.952
6966 measured reflections
2408 independent reflections
1717 reflections with *I* > 2σ(*I*)
*R*_int_ = 0.045

#### Refinement

*R*[*F* ^2^ > 2σ(*F* ^2^)] = 0.058
*wR*(*F* ^2^) = 0.167
*S* = 1.07
2408 reflections
176 parameters
1 restraint
H atoms treated by a mixture of independent and constrained refinement
Δρ_max_ = 0.74 e Å^−3^
Δρ_min_ = −0.35 e Å^−3^
Absolute structure: Flack (1983 ▶), 999 Friedel pairs
Flack parameter: −0.20 (18)

### 

Data collection: *SMART* (Bruker, 1998 ▶); cell refinement: *SAINT* (Bruker, 1998 ▶); data reduction: *SAINT*; program(s) used to solve structure: *SHELXS97* (Sheldrick, 2008 ▶); program(s) used to refine structure: *SHELXL97* (Sheldrick, 2008 ▶); molecular graphics: *SHELXTL* (Sheldrick, 2008 ▶); software used to prepare material for publication: *SHELXTL*.

## Supplementary Material

Crystal structure: contains datablock(s) global, I. DOI: 10.1107/S1600536812005661/su2376sup1.cif

Structure factors: contains datablock(s) I. DOI: 10.1107/S1600536812005661/su2376Isup2.hkl

Supplementary material file. DOI: 10.1107/S1600536812005661/su2376Isup3.cml

Additional supplementary materials:  crystallographic information; 3D view; checkCIF report

## Figures and Tables

**Table 1:** Hydrogen-bond geometry (Å, °)

*D*—H⋯*A*	*D*—H	H⋯*A*	*D*⋯*A*	*D*—H⋯*A*
O2—H2⋯O1^i^	0.82	1.99	2.751 (4)	155
O2—H2⋯N2^i^	0.82	2.48	3.012 (4)	124
N1—H1⋯O2^ii^	0.90 (1)	2.12 (2)	2.987 (4)	164 (5)

Symmetry codes: (i) 
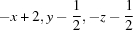
; (ii) 
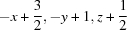
.

**Fig. 1.**
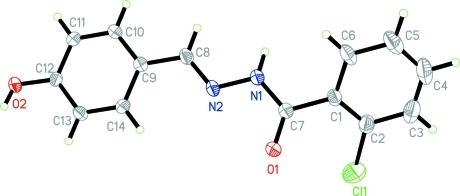
The molecular structure of the title compound, showing the atom labelling scheme. The displacement ellipsoids are drawn at the 30% probability level.

**Fig. 2.**
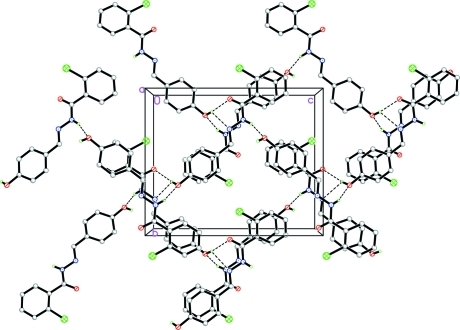
A view along the a axis of the crystal packing of the title compound. The various hydrogen bonds are indicated by dashed lines (see Table 1 for details; C-bound H-atoms have been omitted for clarity)..

## supplementary crystallographic information

### Comment

In recent years, hydrazone compounds have attracted much attention due to their syntheses and crystal structures (Hashemian *et al.*, 2011; Lei, 2011; Shalash *et al.*, 2010). As a continuation of our work on such compounds (Li, 2011*a*,*b*), the author reports herein on the crystal structure of the new title hydrazone compound.

The molecule of the title compound (Fig. 1) exists in a *trans* conformation with respect to the methylidene unit. The dihedral angle between the (C1–C6) and (C9–C1) benzene rings is 37.6 (3)°.

In the crystal, O–H···O, O–H···N, and N–H···O hydrogen bonds leads to the formation of a three-dimensional network (Table 1, Fig. 2).

### Experimental

A mixture of 4-hydroxybenzaldehyde (0.122 g, 1 mmol) and 2-chlorobenzohydrazide (0.171 g, 1 mmol) in 30 ml of methanol containing few drops of acetic acid was refluxed for about 1 h. On cooling to room temperature, a solid precipitate was formed. The solid was filtered and then recrystallized from methanol. Colourless crystals, suitable for X-ray diffraction analysis, were obtained by slow evaporation of a solution of the title compound in methanol.

### Refinement

H atom H1 was located in a difference Fourier map and was freely refined. The remaining H-atoms were positioned geometrically and refined using a riding model: O–H = 0.82 Å, C–H = 0.93 Å, with *U*_iso_(H) = 1.5*U*_eq_(O) and = 1.2*U*_eq_(C).

### Crystal data

**Table:**

C_14_H_11_ClN_2_O_2_	*F*(000) = 568
*M**_r_* = 274.70	*D*_x_ = 1.411 Mg m^−^^3^
Orthorhombic, *P*2_1_2_1_2_1_	Mo *K*α radiation, λ = 0.71073 Å
Hall symbol: P 2ac 2ab	Cell parameters from 1608 reflections
*a* = 7.627 (3) Å	θ = 2.2–24.3°
*b* = 11.859 (2) Å	µ = 0.29 mm^−^^1^
*c* = 14.297 (2) Å	*T* = 298 K
*V* = 1293.2 (5) Å^3^	Block, colourless
*Z* = 4	0.18 × 0.17 × 0.17 mm

### Data collection

**Table:**

Bruker SMART CCD area-detector diffractometer	2408 independent reflections
Radiation source: fine-focus sealed tube	1717 reflections with *I* > 2σ(*I*)
Graphite monochromator	*R*_int_ = 0.045
ω scans	θ_max_ = 25.5°, θ_min_ = 2.2°
Absorption correction: multi-scan (*SADABS*; Sheldrick, 1996)	*h* = −9→8
*T*_min_ = 0.949, *T*_max_ = 0.952	*k* = −14→9
6966 measured reflections	*l* = −17→17

### Refinement

**Table:**

Refinement on *F*^2^	Secondary atom site location: difference Fourier map
Least-squares matrix: full	Hydrogen site location: inferred from neighbouring sites
*R*[*F*^2^ > 2σ(*F*^2^)] = 0.058	H atoms treated by a mixture of independent and constrained refinement
*wR*(*F*^2^) = 0.167	*w* = 1/[σ^2^(*F*_o_^2^) + (0.0863*P*)^2^ + 0.1245*P*] where *P* = (*F*_o_^2^ + 2*F*_c_^2^)/3
*S* = 1.07	(Δ/σ)_max_ < 0.001
2408 reflections	Δρ_max_ = 0.74 e Å^−^^3^
176 parameters	Δρ_min_ = −0.35 e Å^−^^3^
1 restraint	Absolute structure: Flack (1983), 999 Friedel pairs
Primary atom site location: structure-invariant direct methods	Flack parameter: −0.20 (18)

### Special details

**Table:**

Geometry. All e.s.d.'s (except the e.s.d. in the dihedral angle between two l.s. planes) are estimated using the full covariance matrix. The cell e.s.d.'s are taken into account individually in the estimation of e.s.d.'s in distances, angles and torsion angles; correlations between e.s.d.'s in cell parameters are only used when they are defined by crystal symmetry. An approximate (isotropic) treatment of cell e.s.d.'s is used for estimating e.s.d.'s involving l.s. planes.
Refinement. Refinement of *F*^2^ against ALL reflections. The weighted *R*-factor *wR* and goodness of fit *S* are based on *F*^2^, conventional *R*-factors *R* are based on *F*, with *F* set to zero for negative *F*^2^. The threshold expression of *F*^2^ > σ(*F*^2^) is used only for calculating *R*-factors(gt) *etc*. and is not relevant to the choice of reflections for refinement. *R*-factors based on *F*^2^ are statistically about twice as large as those based on *F*, and *R*- factors based on ALL data will be even larger.

### Fractional atomic coordinates and isotropic or equivalent isotropic displacement parameters (Å^2^)

**Table:**

	*x*	*y*	*z*	*U*_iso_*/*U*_eq_	
Cl1	0.7790 (2)	1.15240 (11)	0.03121 (11)	0.0961 (6)	
N1	0.8701 (5)	0.7962 (3)	0.0532 (2)	0.0446 (9)	
N2	0.8920 (5)	0.7314 (3)	−0.0268 (2)	0.0442 (8)	
O1	0.9755 (4)	0.9473 (2)	−0.02416 (18)	0.0479 (8)	
O2	0.8755 (4)	0.3362 (2)	−0.32680 (17)	0.0439 (7)	
H2	0.9466	0.3582	−0.3657	0.066*	
C1	0.8856 (5)	0.9734 (3)	0.1347 (3)	0.0405 (10)	
C2	0.8308 (6)	1.0829 (4)	0.1335 (3)	0.0521 (12)	
C3	0.8123 (6)	1.1435 (5)	0.2166 (4)	0.0698 (15)	
H3	0.7742	1.2180	0.2156	0.084*	
C4	0.8506 (8)	1.0924 (6)	0.2987 (4)	0.0780 (18)	
H4	0.8395	1.1328	0.3541	0.094*	
C5	0.9046 (7)	0.9840 (6)	0.3022 (3)	0.0729 (16)	
H5	0.9287	0.9510	0.3598	0.087*	
C6	0.9240 (6)	0.9228 (5)	0.2230 (3)	0.0551 (12)	
H6	0.9621	0.8484	0.2260	0.066*	
C7	0.9150 (5)	0.9061 (3)	0.0472 (3)	0.0368 (9)	
C8	0.8585 (5)	0.6256 (3)	−0.0187 (3)	0.0408 (9)	
H8	0.8278	0.5966	0.0395	0.049*	
C9	0.8678 (5)	0.5508 (3)	−0.0988 (2)	0.0375 (9)	
C10	0.8151 (6)	0.4403 (3)	−0.0916 (3)	0.0430 (10)	
H10	0.7773	0.4128	−0.0341	0.052*	
C11	0.8172 (6)	0.3700 (3)	−0.1673 (3)	0.0433 (10)	
H11	0.7812	0.2955	−0.1607	0.052*	
C12	0.8724 (5)	0.4086 (3)	−0.2536 (2)	0.0335 (8)	
C13	0.9259 (5)	0.5197 (3)	−0.2627 (3)	0.0387 (9)	
H13	0.9637	0.5465	−0.3204	0.046*	
C14	0.9231 (5)	0.5905 (3)	−0.1863 (3)	0.0413 (10)	
H14	0.9581	0.6652	−0.1929	0.050*	
H1	0.802 (6)	0.767 (4)	0.098 (3)	0.080*	

### Atomic displacement parameters (Å^2^)

**Table:**

	*U*^11^	*U*^22^	*U*^33^	*U*^12^	*U*^13^	*U*^23^
Cl1	0.1370 (15)	0.0600 (8)	0.0912 (11)	0.0223 (9)	0.0056 (10)	0.0157 (8)
N1	0.062 (2)	0.0378 (19)	0.0339 (18)	−0.0073 (18)	0.0079 (17)	−0.0053 (14)
N2	0.062 (2)	0.0404 (19)	0.0304 (17)	−0.0049 (17)	0.0069 (18)	−0.0085 (15)
O1	0.069 (2)	0.0401 (15)	0.0349 (14)	−0.0095 (14)	0.0108 (14)	−0.0016 (13)
O2	0.063 (2)	0.0377 (15)	0.0310 (14)	−0.0025 (15)	0.0058 (13)	−0.0083 (12)
C1	0.040 (2)	0.043 (2)	0.038 (2)	−0.007 (2)	0.0070 (18)	−0.0085 (18)
C2	0.054 (3)	0.047 (3)	0.055 (3)	−0.010 (2)	0.011 (2)	−0.012 (2)
C3	0.061 (3)	0.057 (3)	0.091 (4)	−0.013 (3)	0.025 (3)	−0.027 (3)
C4	0.074 (4)	0.094 (5)	0.066 (4)	−0.027 (4)	0.015 (3)	−0.041 (3)
C5	0.078 (4)	0.102 (5)	0.039 (3)	−0.013 (4)	−0.003 (2)	−0.013 (3)
C6	0.057 (3)	0.076 (3)	0.032 (2)	−0.010 (2)	0.0049 (19)	−0.014 (2)
C7	0.039 (2)	0.038 (2)	0.033 (2)	−0.0001 (18)	0.0004 (17)	−0.0018 (17)
C8	0.048 (2)	0.044 (2)	0.0309 (19)	−0.002 (2)	−0.0013 (19)	−0.0076 (17)
C9	0.042 (2)	0.037 (2)	0.0332 (19)	0.001 (2)	0.0016 (17)	−0.0013 (17)
C10	0.061 (3)	0.038 (2)	0.0300 (19)	0.002 (2)	0.0041 (18)	0.0006 (17)
C11	0.060 (3)	0.030 (2)	0.040 (2)	0.0002 (19)	0.006 (2)	0.0002 (16)
C12	0.038 (2)	0.0296 (19)	0.0330 (19)	0.0046 (17)	−0.0029 (16)	−0.0027 (15)
C13	0.044 (2)	0.044 (2)	0.0284 (19)	0.0010 (18)	0.0038 (16)	0.0011 (17)
C14	0.054 (3)	0.030 (2)	0.040 (2)	−0.0063 (19)	0.0028 (19)	−0.0012 (17)

### Geometric parameters (Å, º)

**Table:**

Cl1—C2	1.724 (5)	C5—C6	1.353 (6)
N1—C7	1.350 (5)	C5—H5	0.9300
N1—N2	1.388 (4)	C6—H6	0.9300
N1—H1	0.896 (10)	C8—C9	1.450 (5)
N2—C8	1.286 (5)	C8—H8	0.9300
O1—C7	1.222 (5)	C9—C10	1.375 (5)
O2—C12	1.354 (4)	C9—C14	1.402 (5)
O2—H2	0.8200	C10—C11	1.366 (5)
C1—C2	1.364 (6)	C10—H10	0.9300
C1—C6	1.429 (6)	C11—C12	1.382 (5)
C1—C7	1.500 (5)	C11—H11	0.9300
C2—C3	1.396 (7)	C12—C13	1.384 (5)
C3—C4	1.353 (8)	C13—C14	1.377 (5)
C3—H3	0.9300	C13—H13	0.9300
C4—C5	1.350 (9)	C14—H14	0.9300
C4—H4	0.9300		
			
C7—N1—N2	116.9 (3)	O1—C7—C1	122.7 (3)
C7—N1—H1	125 (3)	N1—C7—C1	115.1 (3)
N2—N1—H1	116 (3)	N2—C8—C9	121.1 (4)
C8—N2—N1	116.2 (3)	N2—C8—H8	119.5
C12—O2—H2	109.5	C9—C8—H8	119.5
C2—C1—C6	118.3 (4)	C10—C9—C14	118.3 (3)
C2—C1—C7	122.8 (4)	C10—C9—C8	120.7 (3)
C6—C1—C7	118.8 (4)	C14—C9—C8	120.9 (3)
C1—C2—C3	120.7 (5)	C11—C10—C9	121.3 (4)
C1—C2—Cl1	122.4 (3)	C11—C10—H10	119.3
C3—C2—Cl1	116.9 (4)	C9—C10—H10	119.3
C4—C3—C2	119.1 (5)	C10—C11—C12	120.5 (3)
C4—C3—H3	120.5	C10—C11—H11	119.7
C2—C3—H3	120.5	C12—C11—H11	119.7
C5—C4—C3	121.6 (5)	O2—C12—C11	119.0 (3)
C5—C4—H4	119.2	O2—C12—C13	121.8 (3)
C3—C4—H4	119.2	C11—C12—C13	119.3 (3)
C4—C5—C6	120.9 (5)	C14—C13—C12	120.1 (3)
C4—C5—H5	119.6	C14—C13—H13	120.0
C6—C5—H5	119.6	C12—C13—H13	120.0
C5—C6—C1	119.5 (5)	C13—C14—C9	120.5 (4)
C5—C6—H6	120.3	C13—C14—H14	119.7
C1—C6—H6	120.3	C9—C14—H14	119.7
O1—C7—N1	122.2 (3)		

### Hydrogen-bond geometry (Å, º)

**Table:**

*D*—H···*A*	*D*—H	H···*A*	*D*···*A*	*D*—H···*A*
O2—H2···O1^i^	0.82	1.99	2.751 (4)	155
O2—H2···N2^i^	0.82	2.48	3.012 (4)	124
N1—H1···O2^ii^	0.90 (1)	2.12 (2)	2.987 (4)	164 (5)

Symmetry codes: (i) −*x*+2, *y*−1/2, −*z*−1/2; (ii) −*x*+3/2, −*y*+1, *z*+1/2.

## References

[bb1] Bruker (1998). *SMART* and *SAINT* Bruker AXS Inc., Madison, Wisconsin, USA.

[bb2] Flack, H. D. (1983). *Acta Cryst.* A**39**, 876–881.

[bb3] Hashemian, S., Ghaeinee, V. & Notash, B. (2011). *Acta Cryst.* E**67**, o171. 10.1107/S1600536810052128 PMC3050232 21522678

[bb4] Lei, Y. (2011). *Acta Cryst.* E**67**, o162. 10.1107/S1600536810051913 PMC3050239 21522669

[bb5] Li, X.-Y. (2011*a*). *Acta Cryst.* E**67**, o1798. 10.1107/S160053681102366X PMC3151996 21837171

[bb6] Li, X.-Y. (2011*b*). *Acta Cryst.* E**67**, o2511. 10.1107/S1600536811034623 PMC3200979 22065403

[bb7] Shalash, M., Salhin, A., Adnan, R., Yeap, C. S. & Fun, H.-K. (2010). *Acta Cryst.* E**66**, o3126–o3127. 10.1107/S1600536810045162 PMC3011781 21589430

[bb8] Sheldrick, G. M. (1996). *SADABS* University of Göttingen, Germany.

[bb9] Sheldrick, G. M. (2008). *Acta Cryst.* A**64**, 112–122. 10.1107/S0108767307043930 18156677

